# High prevalence and long duration of nervous system and psychiatric adverse drug reactions in Ugandan patients taking efavirenz 600 mg daily

**DOI:** 10.1093/jac/dky298

**Published:** 2018-08-01

**Authors:** Kay Seden, Daniel Kiiza, Eva Laker, Walter J Arinaitwe, Catriona Waitt, Mohammed Lamorde, Saye Khoo

**Affiliations:** 1 Department of Molecular and Clinical Pharmacology, University of Liverpool, Liverpool, UK; 2 Infectious Diseases Institute, Makerere University, Kampala, Uganda; 3 Advanced Treatment Information Centre (ATIC), Infectious Diseases Institute, Makerere University, Kampala, Uganda; 4 Royal Liverpool University Hospital, Liverpool, UK

## Abstract

**Background:**

Efavirenz-related nervous system or psychiatric adverse drug reactions (ADRs) are conventionally reported to resolve soon after initiation, with incidence of dizziness at 8.5% in large clinical trials. Patients of black ethnicity are genetically at greater risk of elevated efavirenz exposure, which has been linked to nervous system toxicity.

**Patients and methods:**

The current data derive from a prospective longitudinal observational study of adult HIV-positive outpatients taking current antiretrovirals, at three diverse clinics in central Uganda. As part of an interview about medicine use, patients were asked by trained pharmacy technicians to detail current side effects and to rate their severity on a simple visual analogue scale (1–10). Details of the reported ADRs were verified by case note review. Severity and causality of ADRs were rated by the study team using validated tools.

**Results:**

A total of 300 patients taking efavirenz were analysed. Of these, 108 (36%, 95% CI 30.6%–41.7%) were affected by persisting nervous system/psychiatric ADRs (median duration 22 months). Dizziness affected 27.3% (95% CI 22.4%–32.8%) of patients taking efavirenz. Severity of the ADRs was rated by patients at ≥5/10 in 76 (58.5%) cases. In 95 (86%) cases, there was no record of the ADRs in the clinical notes.

**Conclusions:**

Strategies are needed to identify and prioritize patients urgently with persisting efavirenz neurotoxicity for a switch to newer regimens as they become available.

## Introduction

Efavirenz remains a mainstay of first-line HIV therapy in Africa. Efavirenz is metabolized by the cytochrome P450 enzyme CYP2B6, which is subject to genetic polymorphisms, meaning that some individuals may be poor or rapid metabolizers via the CYP2B6 pathway. Higher efavirenz exposure has been associated with a higher prevalence of CYP2B6 polymorphisms, which cause loss of metabolic function.^1^ Patients of black ethnicity were found to have 59% higher efavirenz exposure compared with white patients.^2^ Such CYP2B6 polymorphisms were found in 22%–39% of Ugandans, depending on ethnic group.^3^ A South African study demonstrated that increased exposure to efavirenz in CYP2B6*6 homozygotes was significantly associated with increased CNS side effects; however, symptoms resolved within 1 month in all patients.^1^ Recent WHO guidelines recommend a lower dose of 400 mg efavirenz daily as an alternative to the 600 mg dose based on the ENCORE studies, in which 400 mg was found to improve toxicity, while maintaining efficacy, regardless of genotype.^4^^,^^5^ National treatment guidelines for HIV in Uganda (2016) now allow for substitution of efavirenz with dolutegravir, or a dose reduction of efavirenz in patients with significant adverse events. However, efavirenz 400 mg and dolutegravir are not widely deployed across sub-Saharan Africa. Here we report the prevalence, type, severity and duration of CNS adverse drug reactions (ADRs) in a Ugandan outpatient cohort taking efavirenz 600 mg daily.

## Patients and methods

### Study design/population

These data derive from an ongoing prospective longitudinal observational study of adult Ugandan HIV-positive patients taking current antiretrovirals (ARVs) of any duration, in accordance with national guidelines.^6^ Consecutive eligible patients were recruited. The outpatient study has now recruited 868 patients, and aims to quantify the prevalence, type and harm caused by medication safety issues in patients taking ARVs in Uganda.^7^ Patients were aged ≥18 years, taking current ARVs and attending a study site for routine outpatient HIV care.

Data were collected at three outpatient clinics in central Uganda (national referral, urban general and rural district). Trained pharmacy technicians interviewed patients about their medicines, prior to routine scheduled clinic appointments. Interviews followed standard case report forms, and the same open questions were asked of all patients regardless of regimen, with further closed questions specific to patient answers, and supported by case note review. As part of the interview, all patients were asked to describe current symptoms or side effects they suffered from, and nervous system disorder/psychiatric disorder (NSD/PD) symptoms were not specifically sought in the interview. Case notes for each patient were reviewed by the pharmacy technicians, to determine the presence of ADRs not reported by patients, and record laboratory parameters. If symptoms or side effects were recorded in the case notes, technicians confirmed with the patient whether the symptoms were current. Only current symptoms were recorded. Technicians recorded whether each reported side effect was recorded in the patients’ case notes. Data from the first consecutive 300 patients taking an efavirenz-based regimen are presented.

### Adverse event assessment

Reported side effects, symptoms detected via case note review and laboratory abnormalities were considered by the study team for ADR causality assessment. Patients reporting side effects during the interview were asked to describe the severity of each symptom, using a simple visual analogue scale (VAS) numbered from 1 to 10, with 1 being the mildest and 10 the most severe. All ADRs were classified using the Medical Dictionary for Regulatory Activities (MedDRA). Causality was evaluated using the Liverpool Adverse Drug Reaction Causality Assessment Tool (L-CAT),^8^ by at least two members of the study team, which comprised pharmacists and clinicians. Severity of symptoms were rated using the Division of AIDS (DAIDS) Table for Grading the Severity of Adult and Pediatric Adverse Events (Version 2.1).^9^ ADRs in the MedDRA categories ‘nervous system disorders’ (NSDs) or ‘psychiatric disorders’ (PDs) were evaluated in patients taking an efavirenz-containing ARV regimen at the time of interview.

### Statistical analyses

Risk factors for NSD/PD ADRs were assessed using the χ^2^ test (sex, regimen, WHO clinical stage, ARV backbone agents), *t*-test (age, weight) and Wilcoxon rank sum test (CD4 count) as well as OR via logistic regression (STATA/IC version 13).

### Ethics

Ethics approval was obtained from the sponsors and host institutes at all study sites (ethics reference: RETH000829, HDREC336). All patients gave informed consent prior to interview.

## Results

From a sample of 469 patients taking any ARV regimen, 207 patients had one or more ADR, with a total of 305 ADRs detected. Efavirenz-related ADRs of any type accounted for 181 (59.3%) of all detected ADRs. Of 153 NSD/PD ADRs detected in the sample, 85% were probably or possibly related to efavirenz.

Three hundred patients (64%) from the 469 patient sample were taking a current efavirenz-based regimen (Table 1). Of these 300 patients taking efavirenz, 108 (36%, 95% CI 30.6%–41.7%) were affected by a current NSD/PD, possibly or probably associated with efavirenz (130 efavirenz-related NSD/PD ADRs in total).

**Table 1.** Characteristics of Ugandan patients taking efavirenz, and potential risk factors for NSD/PD ADRs

	Group				
Patient factor	all patients (*n* = 300)	EFV NSD/PD [*n* = 108 (36%)]	no NSD/PD [*n* = 192 (64%)]	*P* value^a^	OR (95% CI)^b^
All, *n* (%)	300 (100)	108 (36)	192 (64)	–	
Age, years, mean (95% CI)	34.88 (33.63–36.14)	33.91 (31.77–36.05)	35.43 (33.88–36.98)	NS	0.99 (0.97–1.0)
Sex, *n* (%)					
F	209	79 (37.8)	130 (62.2)	NS	0.77 (0.46–1.30)
M	91	29 (31.9)	62 (68.1)		
Weight, kg, mean (95% CI)	59.26 (58.03–60.49)	60.27 (58.05–62.48)	58.7 (57.23–60.17)	NS	1.01 (0.99–1.04)
CD4 count, cells/mm^3^, median (IQR)	477.5 (311.5–646)	463 (279–680)	484 (336–645)	NS	0.99 (0.998–1.00)
Regimen, *n* (%)					
TDF/3TC/EFV	262 (87.33)	94 (35.9)	168 (64.1)	NS	0.92 (0.45–1.87)
ZDV/3TC-EFV	37 (12.33)	14 (37.8)	23 (62.2)		
WHO stage, *n* (%)					
1	139 (46.3)	55 (39.6)	84 (60.4)	NS	0.98 (0.76–1.27)
2	94 (31.3)	28 (29.8)	66 (70.2)		
3	45 (15)	14 (31.1)	31 (68.9)		
4	22 (7.3)	11 (50)	11 (50)		

TDF, tenofovir disoproxil fumarate; 3TC, lamivudine; EFV, efavirenz; ZDV, zidovudine; NS, not significant.

a *t*-test was used for means of continuous variables (Wilcoxon rank sum for non-parametric), χ^2^ for categorical variables.

b OR by logistic regression.

Dizziness was by far the most prevalent ADR, accounting for 82 (63%) of the 130 NSD/PD symptoms, and affecting 27.3% (95% CI 22.4%–32.8%) of the 300 patients on efavirenz. Other symptoms were: drowsiness/somnolence (12), headache (11), hypoaesthesia/paraesthesia (9), abnormal dreams (5), blurred vision (4), memory impairment (2), hallucinations, psychosis, taste disturbance, insomnia and anxiety (1 each).

The median duration of the ADRs detected was 22 months (IQR 9–35.3), which for most patients was the full duration of their efavirenz-based regimen. (Median time on efavirenz for the 300-patient sample was 23 months, IQR 11–39.5.)

**Figure 1.**
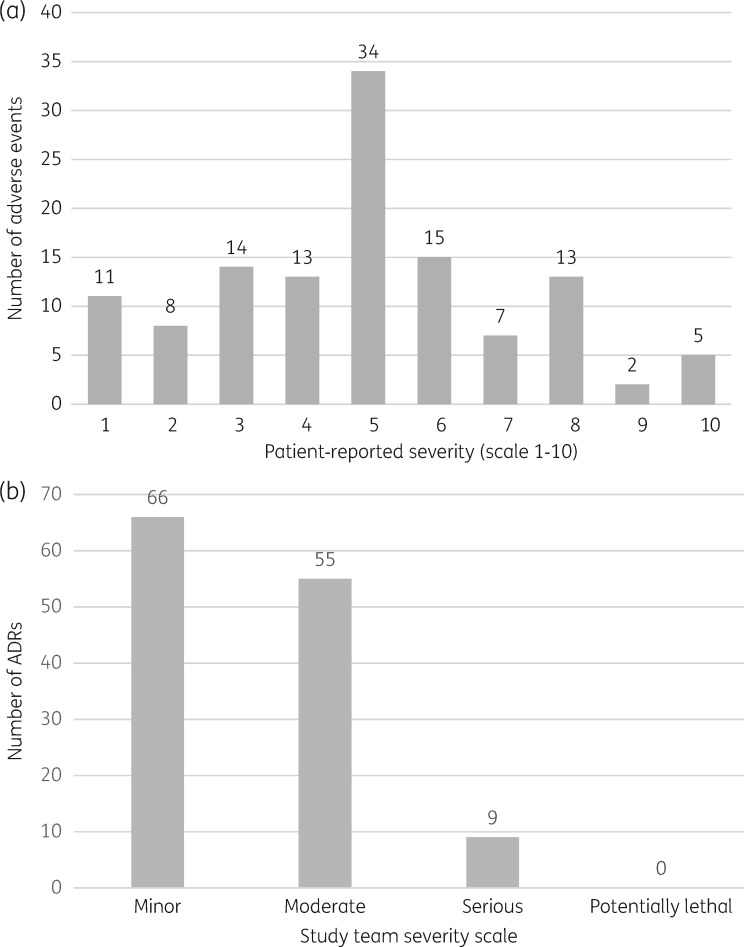
(a) Patient-rated severity of efavirenz-associated NSD/PD ADRs (VAS 1–10). ADRs reported: *n *=* *130, by 108 patients. Severity was reported by patients in 122 cases. (b) Study team severity rating of NSD/PD ADRs related to efavirenz (DAIDS classification).

The severity of the ADRs was rated by patients at ≥5/10 in 76 (58.5%) cases (Figure 1a). All numbers in the VAS were used at least once during the patient report. The study team rated the severity of the ADRs as ‘minor’ in half of the cases (Figure 1b). In 95 (86%) cases, there was no record of the ADRs in the patient's clinical notes (excluding symptoms that were reported at the current clinic visit). Fourteen ADRs reportedly began between the previous clinic visit and the study interview. Of the ADRs not recorded in the case notes, 55 (58%) were considered minor. For the 15 ADRs recorded, about one-quarter were minor.

Age, weight, CD4 count, WHO stage, gender and ARV backbone agents (tenofovir disoproxil fumarate/lamivudine/efavirenz versus zidovudine/lamivudine/efavirenz) were not significantly associated with the risk of ADRs (Table 1).

## Discussion

A high proportion of Ugandan patients taking efavirenz were affected by NSD/PD ADRs (36%), which were unresolved at the time of assessment. Most notably, 75% of NSD/PD symptoms were experienced for at least 9 months (median 22 months), with symptoms ongoing at the point of analysis. This differs considerably from the short-term symptoms reported in the manufacturer’s literature, which generally resolve within 2–4 weeks.^10^ In a systematic review of 42 clinical studies (predominantly white participants), the prevalence of neuropsychiatric adverse events was 29.6%, with dizziness, the most prevalent, affecting 12.8%. Dizziness was more commonly reported in the first 48 weeks of treatment.^11^ In a study of 80 black South African patients initiating efavirenz, 55% reported dizziness. However, all symptoms resolved within 1 month.^1^ The prevalence of dizziness reported by the manufacturer’s summary of product characteristics is 8.5%, from >9000 patients in clinical trials.^10^ We report a prevalence of dizziness at 27.3% in Ugandan patients. Our data may differ from clinical trial data and meta-analyses of such owing to the relative lack of black patients and women included in the study populations.^12^ However, various studies have reported long-term NSD/PD ADRs in a lower proportion of patients taking efavirenz, of ∼2 years duration.^13^^,^^14^

We observed a lack of documentation or reporting of NSD/PD ADR symptoms in the case notes in 86% of evaluable cases. Lack of recognition (and consequently management) of NSD/PD symptoms perpetuates the hidden burden of efavirenz toxicity, and delays appropriate patient management.

It should be noted that our current estimate of NSD/PD prevalence may be conservative, since we did not adequately capture depression, which is likely to be under-recognized by patients and clinicians, and under-reported in this setting. Another limitation of these data is the relatively low patient numbers, particularly men (*n* = 91) evaluated to date.

The VAS used appeared to be well understood by patients in the study, with literacy not being a barrier. The whole range of the scale was used during patient reporting, with a spread around the central value, 5 (Figure 1a). The study team rated half of the ADRs as ‘minor’, demonstrating some discord with the patient experience. Although expert causality assessment for ADRs is essential, the severity of ADRs should take into account the patient experience, particularly in the case of NSDs/PDs.

Patient-rated ADR severity represents a useful screening tool for adverse events such as NSDs and PDs, in which the way quality of life is affected for the individual patient is not always apparent to clinicians. The limiting factor for being able to manage such patients, aside from availability of alternatives, is detection of symptoms. Therefore, active screening with such a tool, followed by clinical assessment and formal quality of life assessment is proposed as a strategy to identify patients eligible to switch from efavirenz 600 mg once daily to reduced-dose efavirenz, or newer agents such as dolutegravir, as they become available in sub-Saharan Africa.
